# Simulation and Structural Optimization of an Eccentric Rotor Extruder Feeding Section

**DOI:** 10.3390/ma18091939

**Published:** 2025-04-24

**Authors:** Jinhui Jiang, Yanhong Feng, Shuo Gao, Wenqiang Yan, Xiaochun Yin, Guizhen Zhang

**Affiliations:** 1 The National Engineering Research Center of Novel Equipment for Polymer Processing, South China University of Technology, Guangzhou 510641, China; 201910100556@mail.scut.edu.cn (J.J.);; 2 Key Laboratory of Polymer Processing Engineering, Ministry of Education, South China University of Technology, Guangzhou 510641, China

**Keywords:** eccentric rotor extruder, discrete element method, feed opening design, feeding section simulation, solid conveying process

## Abstract

The eccentric rotor extruder (ERE) is polymer processing equipment that exhibits excellent processing capabilities for materials with extremely high viscosity, which are difficult to plastically deform and transport efficiently. However, the mass transfer mechanism in the solid conveying section of this new device is fundamentally different from that of traditional extruders, and no related research has been reported. This study uses discrete element method (DEM) simulation technology to model the solid conveying process of the ERE. By visualizing the positive displacement conveying process, and with an analysis of the output parameters, the study clarifies the mass transfer principles and quantifies the conveying capacity, providing guidance for optimizing the extruder design. The simulation results show that the ERE exhibits positive displacement conveying characteristics, with the conveying process achieved by the forward movement of the cavities (closed cavities between the rotor and stator) in a helical manner. However, differences in the dual-cavity (two types of cavities) feeding process and low fill level can lead to significant fluctuations in extrusion output and reduced conveying capacity. To address these issues, an improvement scheme for the dual-cavity feed opening is proposed, with feed openings designed with different opening lengths. Then, by analyzing the particle coordinate data from the simulation output, the conveying capacities of different feed opening structures are quantified and optimized. Finally, experimental and simulation verification results indicate that the optimized structure significantly improves the issues of uneven filling and low fill level, with good correspondence between the simulation and experimental results. Simulation results show that, compared with the original structure, the optimized dual-feed opening structure increases the feed capacity from 3953 particles per cavity to 5132 particles per cavity, an improvement of 29.8%, and it achieves balanced filling between the two cavities. Experimental validation indicates that the UPE4040 output can be increased from 165.3 g/min with structure op-00 to 231.7 g/min with the optimized structure op-05.

## 1. Introduction

The eccentric rotor extruder (ERE) is polymer processing equipment that offers advantages such as low energy consumption, short thermal-mechanical history, high adaptability to materials, and good dispersion and mixing performance [1]. It has demonstrated good performance in processing composite material systems such as a polymer filler system [2,3], polymer blend systems [4,5], and high-performance polymers like ultra-high molecular weight polyethylene [6,7,8], which are difficult to process.

**Figure 1:**
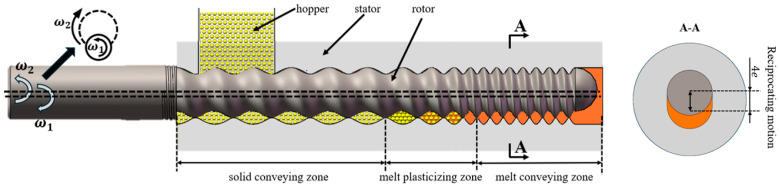
Schematic diagram of the eccentric rotor extruder structure.

The structure of the ERE is shown in Figure 1 [5]. The ERE primarily consists of an intermeshing eccentric rotor and a stator. The rotor rotates around its own axis while also rolling around the stator’s axis within the inner cavity of the stator. The material is advanced within the helical closed cavities formed between the stator and rotor. During this advancement processes, such as compaction, degassing, melting, melt transportation, and mixing of the material are achieved [9]. ERE’s efficient mass transfer effect is based on the sealing of its cavities, enabling the forced conveying of material within. However, during the feeding process, the cavities are not closed, allowing material exchange between the inside and outside of the cavities. This exchange leads to variations in the fill level within the cavities, thereby affecting the mass transfer efficiency of the ERE. No relevant research has been reported on how the feeding process influences the mass transfer in the ERE.

For the development and research of new equipment, relying solely on experimental data accumulation and experience would be very time-consuming and labor-intensive. Thanks to advancements in computer technology, the discrete element method (DEM) has become a powerful tool to simulate particle motions in recent years. The DEM was introduced by Cundall [10]; by considering the interactions between particles and between particles and walls, it can predict the flow behavior of solid particles with relative accuracy. As a result, it has been widely applied to powder transport systems, such as screw conveyer [11,12,13,14,15,16,17], feeder [18,19,20], drums [21,22,23,24], etc. [25,26,27], which helps analyze particle flow patterns, predict equipment performance, and further study on the underlying mechanisms, providing guidance for structural optimization. The Altair® EDEM™ 2023 (discrete element method (DEM) software) was selected, which is widely used to simulate particle motions using discrete elements [25,28,29,30], and it was used in this study to investigate the solid conveying process during the feeding of the ERE.

The DEM simulation technique is effective for studying solid transfer in conveying equipment, providing particle parameters that are hard to obtain through experiments and enabling the quantification of transfer efficiency. For example, Owen and Cleary [31] used DEM simulations to predict the impact of operating conditions, such as inclination, speed, and fill level, on screw conveyor performance. Kretz et al. [19] analyzed different screw types using DEM simulations, obtaining mass flow rates over time by tracking particles that fell out of the screw cylinder, revealing how screw structures affect conveying stability. DEM also allows for the visualization of mass transfer, aiding in the capture of particle flow characteristics. Moysey and Thompson [16] used DEM to analyze particle behavior near an extruder’s feed opening, finding periodic fluctuations and recirculating flow patterns.

By using DEM simulations to compare the impact of structural changes on equipment conveying performance, device structure can be optimized cost-effectively, and optimization solutions can be quickly validated. For example, Li et al. [32] studied the effect of asymmetric screws on extrusion performance, finding that they improved extrusion by reducing bridging and enhancing particle flow. Dun et al. [33] simulated the impact of feeder inclination angle and length on performance, quantifying it through extrusion stability and output, and optimizing parameters via response surface analysis. Fu et al. [34] analyzed how strike angle affects drilling tool performance, providing important insights for tool design and optimization. These examples show that DEM simulation is well suited for this study. Since current research on the ERE mainly focuses on its melt conveying section, while studies on the solid conveying process are still lacking, this study aimed to reveal the feeding mechanism of the ERE through DEM simulation and visualization. Then, a structural improvement plan was proposed, and the effectiveness of the structural optimization was validated, aiming to improve the delivery efficiency of the ERE solid conveying process and address the issue of cavity filling imbalance, which helps shorten the length-to-diameter ratio of the ERE structure, reduce energy consumption, and decrease product defects.

This paper will leverage discrete element method (DEM) simulation technology to visualize the feeding process of ERE and efficiently obtain real-time particle-related parameters during the feeding process. Through the visualization and analysis of the solid conveying process, the flow pattern of the positive displacement conveying process will be elucidated, and the key processes and structures affecting the mass transfer efficiency will be identified. Based on the flow pattern, corresponding design improvement proposals will be made. By analyzing the particle parameter data from the simulation results, the conveying performance will be quantified, compared, and optimized. Finally, the feasibility of the optimized structural design will be validated through experiments.

## 2. Materials and Methods

**Figure 2:**
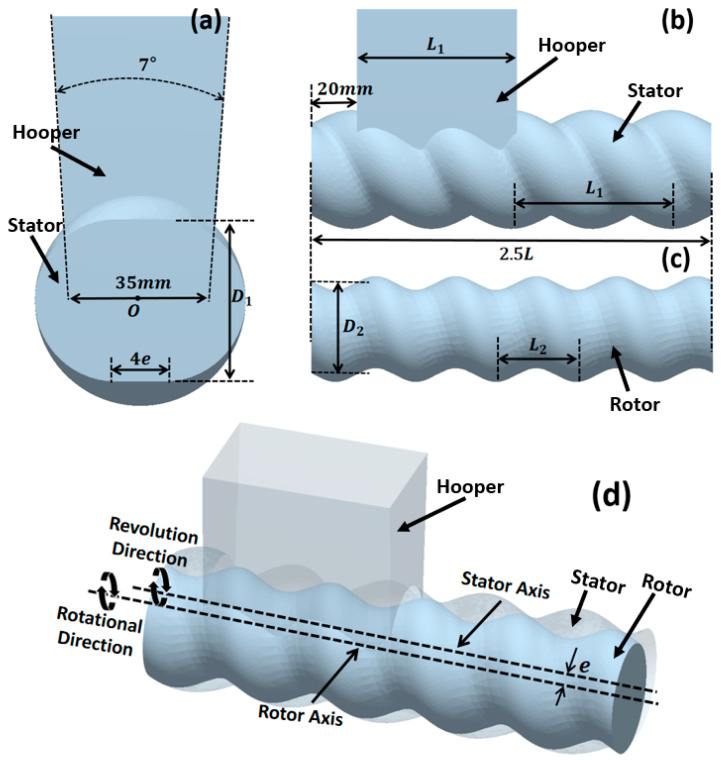
ERE solid conveying section geometry model: (**a**) stator and hopper cross-sectional dimensions, (**b**) stator and hopper axial dimensions, (**c**) rotor dimensions, (**d**) model perspective view.

To simulate the solid feeding process of the ERE, a geometric model was constructed (as shown in Figure 2), with parameters referring to the solid conveying section structure of the ERE-40 (Siiico Technology Co., Ltd., Guangzhou, China). The specific geometric structure and operating parameters are shown in Table 1. The model includes three parts: the hopper, stator, and rotor, as shown in Figure 2. The rotor and stator are in an intermeshing state, and the position and shape of the hopper are shown in Figure 2a,b.

**Figure 3:**
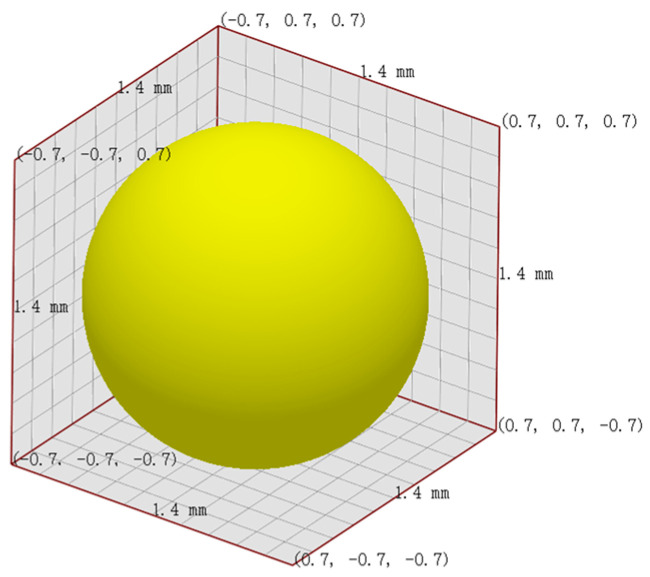
Schematic diagram of particle shape and size.

This study uses Altair^®^ EDEM™ 2023 (DEM Solutions Ltd., Edinburg, UK) for discrete element simulation, with the Hertz–Mindlin (no slip) contact model [35] employed to simulate interactions between particles and between particles and the ERE device. The particle and geometric material parameters are based on plastic (based on DEM solutions engineering experience) and steel [36]. Considering the computational power of the computer and approximating the particle size parameters of ultrahigh-molecular weight polyethylene UPE4040 (Shanghai Chemical Industry Research Institute, Shanghai, China), which has a weight-average molecular weight of 6.5 million, has a density of 0.93 g/cm^3^ and a loose bulk density of 0.42 g/cm^3^, the particle size is 150–200 μm, the particle diameter was set to 1.4 mm, as shown in Figure 3. The simulation time step was set to 25% of the Rayleigh time step, and the parameter settings are shown in Table 2.

## 3. Research Results and Analysis

### 3.1. Mass Transfer Process in the Solid Conveying Section of the ERE and Problem Analysis

**Figure 4:**
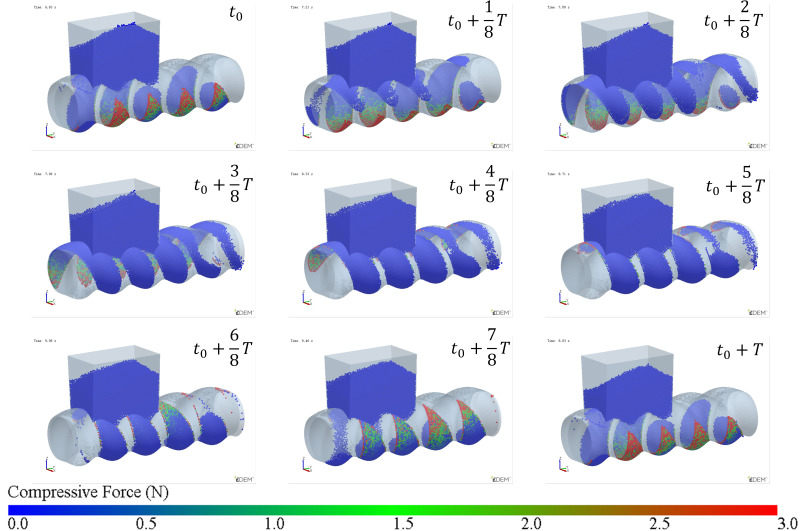
Mass transfer and force analysis of particles in the ERE solid conveying section (cycle time T = 3 s).

The simulation results of particle mass transfer and forces in the solid conveying section of the ERE are shown in Figure 4 (see Video S1). From Figure 4, it can be observed that, due to the meshing between the rotor and stator, several closed cavities are formed, which are not interconnected. Particles are trapped in these cavities, and as the rotor rotates, these cavities move axially in a helical motion, forcing the particles inside to follow the movement. When the rotor rotates at a constant speed, within one cycle, the cavities move forward by one stator pitch, and the particles inside move forward by the same pitch. The axial speed of particles in the ERE is primarily determined by the pitch and rotational speed of the ERE and is largely unaffected by other factors such as particle properties. The simulation also shows that the filling levels of the cavities are not consistent. Along the axial direction, there are alternating cavities with high and low filling levels. The difference in filling levels leads to uneven forces, with particles in fuller cavities experiencing higher forces. The filling level differences may cause a sharp rise in pressure in the compression section due to further compression of the fuller cavities, while the pressure in emptier cavities remains lower. The imbalance in the dual-cavity (two types of cavities) feed flow causes uneven forces on the rotor. Currently, in the ERE, by increasing the gap between the rotor and the stator, particles in the cavities can flow through the gap and interact with each other, allowing solid particles to reflux under pressure and balance the cavity-filling degree. However, this sacrifices the positive displacement conveying capacity, thereby reducing the conveying efficiency. This paper explores methods to balance and increase the number of particles conveyed in the cavity without increasing the gap.

**Figure 5:**
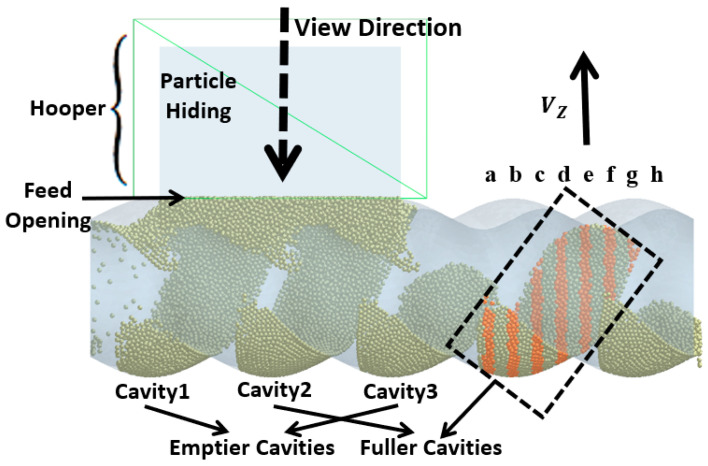
Simulation results for ERE solid conveying section and particles: (a–h) highlighted particles (highlight in orange) at different axial positions.

**Figure 6:**
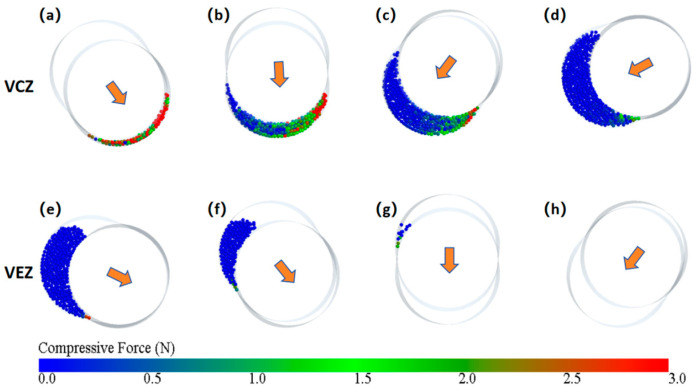
Force analysis diagrams of particles at different axial positions: (**a**–**h**) corresponding to the highlighted particles in Figure 5 (the arrow indicates the direction of rotor movement at the present cross-sectional view).

To analyze how the rotation of the rotor drives the movement of particles inside the cavity, the force variation of the particles within the cavity (the cavity within the black dashed-line square in Figure 5) is analyzed. The forces acting on the particles at different axial positions are shown in Figure 6a–h, with the cross-sectional positions indicated in Figure 5a–h (highlight particles).

When the eccentric rotor performs specific orbital and rotational motions, its sectional circle rotates while simultaneously undergoing a reciprocating translational movement along the stator’s long groove. The direction of this translational motion is indicated by the orange arrow in Figure 6. From the changes in the direction of the arrows at different cross-sectional positions, in the front half of the cavity (Figure 6a–d), the arrow direction points toward the region where the particles are located. The rotor’s movement reduces the volume of the cavity and compresses the particles, forming the volume compression zone (VCZ), where the particles experience greater force. In the latter half of the cavity (Figure 6e–h), the arrow direction is away from the region where the particles are located. The rotor’s movement causes the cavity’s volume to expand, forming the volume extension zone (VEZ), where the particles experience less force. The particles in the VCZ continuously flow out under the extrusion effect. Due to the sealed nature of the cavity, the particles flow toward the VEZ, and the continuous expanding of the VEZ maintains its relatively low-pressure state. Therefore, it can be concluded that the driving force for the ERE particle movement primarily comes from the compression induced by the rotor movement in the VCZ. Particles that flow out of the VCZ move through the enclosed cavities and are guided toward the VEZ, continuously driving the particles to spiral forward.

### 3.2. Feeding Process in the Solid Conveying Section of the ERE and Problem Analysis

In the process of transporting particles through the enclosed cavities, the number of particles inside the cavities remains constant. The variation in the number of particles occurs in the feeding zone, where the cavities is not closed, and particles can flow into the cavities from the hopper or flow back from the cavities to the hopper through the gap (feed opening) above the stator. To analyze how the filling differences in the cavities during the feeding process are caused, the particles above the feed opening are hidden, and the variation in the particle movement speed along the gravity direction (Vz) near the feed opening (as shown in Figure 5) is observed.

**Figure 7:**
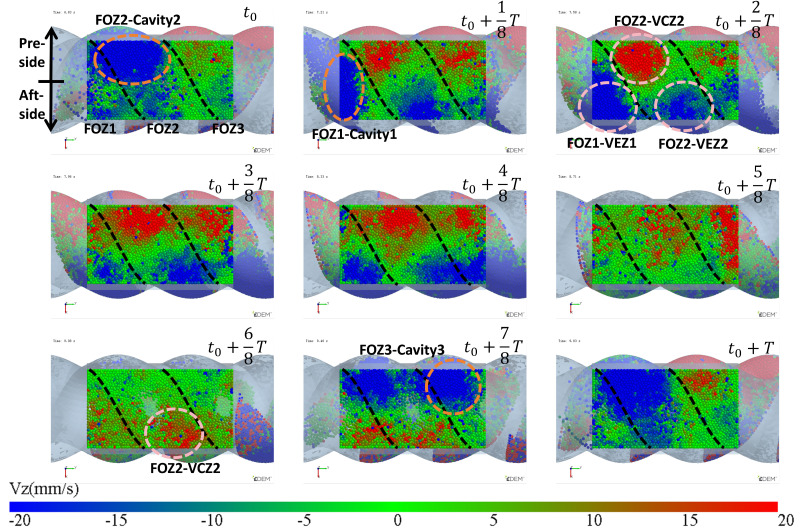
Variation of the vertical velocity (Vz) of particles near the feed opening with time (the regions FOZ1, FOZ2, and FOZ3 are separated by black dashed lines).

**Figure 8:**
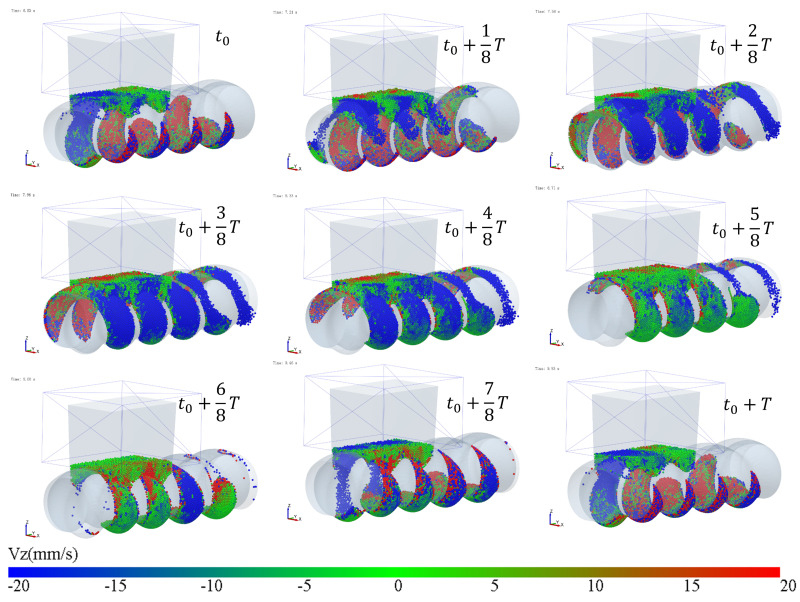
Variation of the vertical velocity (Vz) of particles with time.

The variation in velocity is shown in Figure 7 and Figure 8 (see Video S2). Particles only flow into the cavities when they move near the feed opening, and the feed opening becomes a gap in the enclosed cavities. Since the cavities spiral forward along a fixed trajectory, and the position of the feed opening, which can become its gap, is also fixed (feed opening zone, FOZ). As shown in Figure 7, the black dashed lines divide the feed opening regions (Figure 7 (t_0_): FOZ1, FOZ2, and FOZ3) corresponding to the three cavities (Figure 5: Cavity1, Cavity2, and Cavity3). Because cavities rotate in a clockwise direction along the axial direction, they will sequentially connect with the feed opening from both the upper and lower sides (pre-side and aft-side). And the process of feeding from the upper and lower sides differs. The simulation results show that the feeding of the three cavities (blue particles in Figure 7) is not synchronous.

The feeding process can be divided as follows.

In Figure 7 (t_0_ + 7/8T, t_0_ + T, t_0_ and t_0_ + 1/8T), particles flow into the cavities through the pre-side feed openings. It can be observed that the filling processes of different cavities vary. The feed opening zone of Cavity2 (FOZ2) is the largest, resulting in the highest feeding rate (as t_0_: FOZ2–Cavity2), and the pre-side cavity is completely filled as early as t_0_ + 1/8T. In contrast, the feed opening zone of Cavity1 (FOZ1) is the smallest, leading to a slower feeding rate (as t_0_ + 1/8T: FOZ1–Cavity1), and the pre-side cavity is not fully filled until t_0_ + 2/8T. The feed opening zone of Cavity3 (FOZ3) is of intermediate size, providing a relatively fast filling rate (as t_0_ + 7/8T: FOZ3–Cavity3). Additionally, Cavity 3 has a higher initial filling degree (originating from the previous feeding cycle at the Cavity1 position), and its pre-side cavity is fully filled by t_0_ + T.

In Figure 7 (t_0_ + 2/8T to t_0_ + 5/8T), particles flow into the cavities through the aft-side feed openings. The feeding rate varies with the expansion of the VEZ in the aft-side cavity. Since the VEZs of Cavity1, 2, and 3 expand at the same rate on the aft-side, the feeding rates of Cavity1 and 2 (as t_0_ + 2/8T: FOZ1–VEZ1 and FOZ2–VEZ2) are comparable. However, the feeding rate of Cavity 3 is relatively lower for the same reason as the reduced feeding rate observed in the aft-side cavity feeding process mentioned above.

The simulation results also show that, in addition to feeding, there is back feeding (represented by red particles). Back feeding can likewise be divided into pre-side and aft-side back feeding. Pre-side back feeding occurs after the pre-side cavities are filled, in Figure 7 (t_0_ + 2/8T to t_0_ + 5/8T), as the compression zones (VCZ) of Cavity1, 2, and 3 compress, particles are squeezed out of the pre-side cavities, with some flowing back into the hopper and some flowing into the aft-side cavities. Since the size of the feed opening zone: FOZ2 > FOZ3 > FOZ1, the back feeding rates follow the order: Cavity2 > Cavity3 > Cavity1, with Cavity 2 exhibiting the highest back feeding rate (as t_0_ + 2/8T: FOZ2–VCZ2).

As aft-side back feeding, in Figure 7 (t_0_ + 6/8T to t_0_ + T), the volume (VEZ) of the aft-side cavity continues to increase, but the feed rate does not grow synchronously with the volume increase. Since the cavity moves in a spiral, as the aft-side cavity (VEZ) expands in the vertical upward direction, the feeding, which relies on gravity, cannot effectively fill this portion of the volume by the feed opening. When the cavity completely passes through the feed opening, the VCZ begins to connect with the FOZ. However, due to the gap at the advancing face of the cavity, particles inside the cavity cannot fully advance with the cavity. Under the compressive action of the rotor, some of the particles flow back (as t_0_ + 6/8T: FOZ2 − VCZ2).

Thus, it can be concluded that, because the movement trajectories of Cavity1 and Cavity3 are the same, their feeding processes are similar. However, the movement trajectory of Cavity2 is different, and its feeding process is therefore distinct from the others. Even though the feeding area of Cavity2 (FOZ2) is equal in size to the feeding areas of Cavity1 and Cavity3 (FOZ1 + FOZ3), Due to the differences in the configuration conditions along the movement trajectories of the cavities, the filling levels of Cavity1 and Cavity3 (as shown by the fuller cavities in Figure 5) will ultimately be significantly higher than that of Cavity2 (as shown by the emptier cavity in Figure 5).

### 3.3. Dual-Cavity Feed Opening Adaptation Design

From the above analysis, it can be concluded that the differences in filling levels between different cavities are due to the different motion trajectories of the cavities during feeding, which result in the cavities feeding from different feed opening regions. These differences in feed opening regions cause variations in the feeding process. To solve the problem of filling imbalance in the cavities, the same feed opening structure should be configured along the motion trajectories of the cavities (dual-cavity balanced design (DCBD)). Traditional feed openings are designed with regular shapes (rectangular or circular), which do not allow for the effective configuration of identical feed regions along the motion trajectories of different cavities. The motion trajectories of the cavities are two distinct paths: one corresponds to cavities with higher filling levels (such as Cavity1 and Cavity3), and the other corresponds to cavities with lower filling levels (such as Cavity2).

**Figure 9:**
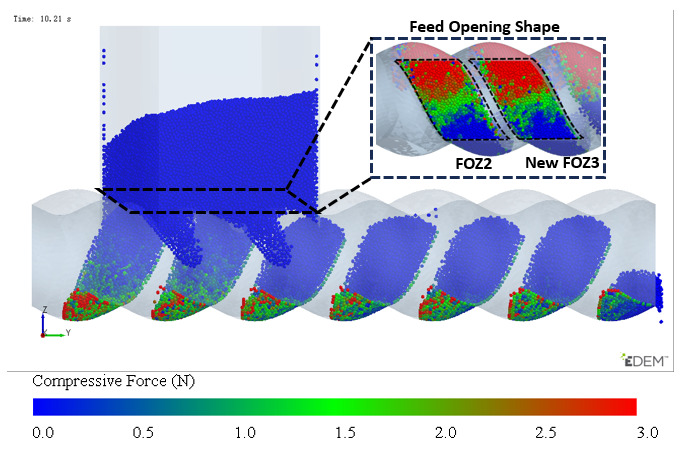
Dual-cavity feed opening adaptation design scheme and feeding effect.

Configured with identical feed opening structures, as shown in Figure 9, the region enclosed by the dashed lines represents the new structure, where the original structure (as shown in Figure 7) has removed FOZ1 and enlarged FOZ3, making New FOZ3 = FOZ2. The area between the two feed openings is the shared feed region, which will have different feeding effects on the dual-cavity, thereby affecting the filling balance, so it is enclosed. Since the motion trajectories of the dual-cavity are separated by 1/2 stator pitch, the two feed openings are axially offset by 1/2 a stator pitch. Under the current feed opening configuration conditions, the simulation results are shown in Figure 8. By observing the filling conditions of the cavities after feeding, it can be seen that the feeding effect for the dual-cavity is comparable. From this, it can be concluded that the DCBD is effective in solving the problem of uneven filling between the two types of cavities. However, the structure shown in Figure 9 is adapted for the feed opening of Cavity2, and its filling level is similar to that of the cavities with lower filling, which leads to a relatively low filling level. Therefore, the feed opening for Cavity2 needs further optimization.

Since DCBD ensures a consistent feeding process for dual-cavity, the subsequent analysis will focus on only one of dual-cavity (e.g., Cavity2 in Figure 5), and the analysis of the other dual-cavity will not be repeated. The reason for the different filling degrees of Cavity1 and Cavity2 in the original structure can be found in Appendix A.

### 3.4. Dynamic Tracking of Cavities and Efficient Statistics of Internal Particle Quantity

From the Vz change diagram at the feed opening (Figure 7), it can be observed that, during the process where the cavity connects to the feed opening, not only feeding occurs but also back feeding, leading to complex changes in the number of particles inside the cavity. To efficiently and accurately track the changes in the filling level inside the cavity and provide more detailed information for identifying the reasons for low filling levels and validating optimization effects, it is necessary to track the cavity as it spirals forward and determine the method for calculating its boundaries. Using the EDEMpy 0.1.2 interface and Python 3.7.6 programming, the process of counting particles within the cavity boundaries can be automated (see Python code S1). This will enable efficient and accurate statistics on the number of particles inside the cavity, providing valuable insights into the filling behavior and the effects of any optimization strategies.

**Figure 10:**
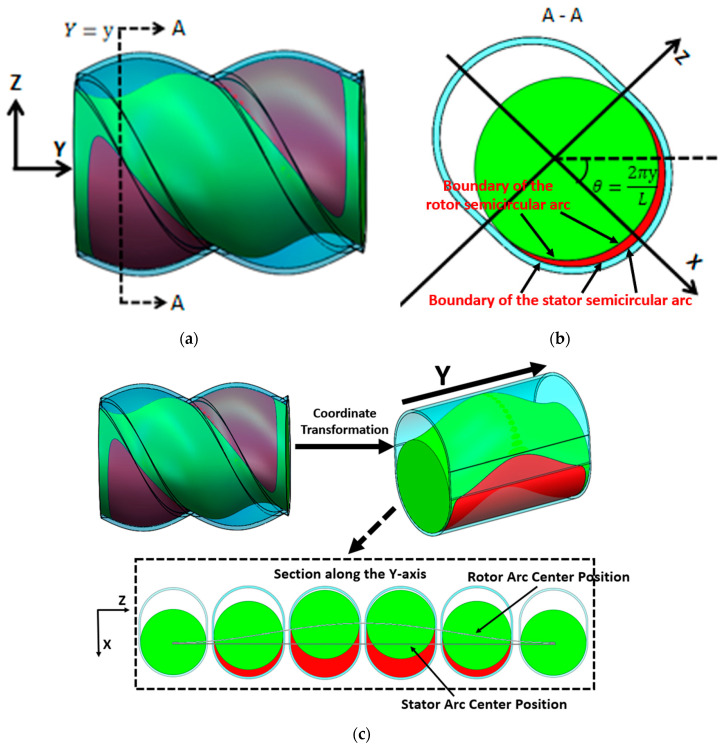
Schematic diagram of the cavity (the rotor is indicated in green, the stator in blue, and the cavity region in red): (**a**) three-dimensional structure, (**b**) cross-sectional structure, (**c**) schematic of cavity boundary calculation.

The position of the cavity moves forward along a fixed spiral path over time, and it needs to be dynamically tracked. The forward speed of the cavity is *wL*, and the rotational speed is 2*πw*. The cavity’s shape and structure are shown in the red area of Figure 10a, with an axial distribution range that is complex in three dimensions. The cross-section along the Y-axis of the cavity is depicted in Figure 10b, which reveals distinct structural features. The cavity’s cross-sectional area is located between the semi-circular arcs of the stator and rotor. The longitudinal direction of the stator slot is shown in Figure 10b, and the angle between it and the horizontal direction is denoted as *θ*. This angle is only related to the pitch and axial position of the cavity. The distance between the center of the stator’s semi-circular arc and the central point remains constant, whereas the center distance of the rotor’s semi-circular arc varies with time and axial position. Continuous tracking of the variation in the rotor’s arc center distance is required to accurately capture the cavity’s position and boundaries at any given time.

To simplify subsequent calculations, a new coordinate system is established on different axial cross-sections, as shown in Figure 10b. The X-axis of the new coordinate system is oriented along the longitudinal direction of the stator slot. The angle between the X-axis and the horizontal line increases linearly along the axial direction. The coordinates of the particles at different axial positions are then pre-processed as follows:

(1) $$x_{1}=x\cdot cos\frac{2\pi \cdot y}{L}-z\cdot sin\frac{2\pi \cdot y}{L}\;z_{1}=z\cdot cos\frac{2\pi \cdot y}{L}-x\cdot sin\frac{2\pi \cdot y}{L}$$

In the new coordinate system, the boundary of the stator semicircular arc can be expressed as follows:

(2) $$x=2e+\sqrt{R^{2}-z^{2}}\;(-R<z<R),$$

The position of the rotor’s semicircular arc changes with time and axial position, and its boundary is as follows:

(3) $$x=2e\cdot \cos\left(2\pi w\left(t+t_{0}\right)+\frac{2\pi \cdot y}{L}\right)+\sqrt{R^{2}-z^{2}}\;(-R<z<R),$$

The condition for determining whether a particle is located within the cavity is as follows:

(4) $$\left\{\begin{array}{c} 2e\cdot \cos\left(2\pi w\left(t+t_{0}\right)+\frac{2\pi \cdot y}{L}\right)+\sqrt{R^{2}-z^{2}}<x_{1}<2e+\sqrt{R^{2}-z^{2}}\\ y_{0}+w\cdot L\cdot t<y<y_{0}+w\cdot L\cdot t+L\\ -R<z_{1}<R \end{array}\right.,$$

where the following applies: *w* is the revolution velocity [r/s]; *L* is the stator pitch [mm]; *y*_0_ is the axial starting position of the cavity [mm]; *θ* is the angle between longitudinal direction of the stator slot and the horizontal line [rad]; *e* is eccentricity [mm]; *x*, *y*, and *z* are the particle coordinates [mm]; *x*_1_ and *z*_1_ are the new particle coordinates [mm]; and *t*_0_ is the starting time [s].

### 3.5. Analysis of the Variation in the Number of Particles Inside the Cavity

**Figure 11:**
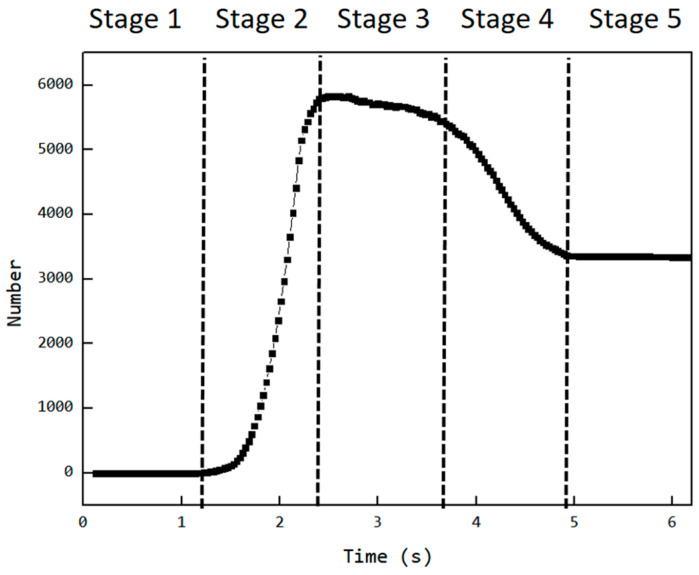
Variation in the number of particles inside Cavity2 with time.

**Figure 12:**
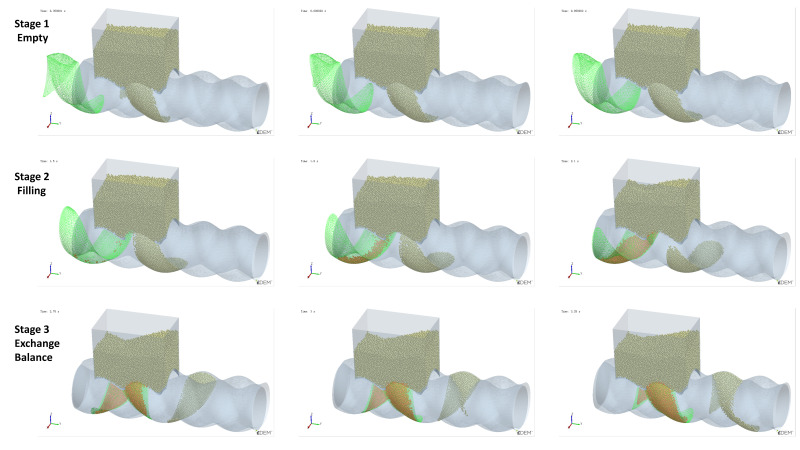

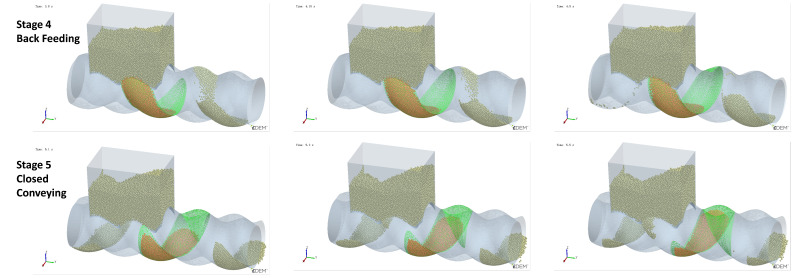
Relative position of the cavity and feed opening at different stages and their filling levels (green mesh indicates the cavity location, while orange particles denote those within the cavity).

As mentioned earlier, Cavity2 was once filled during the feeding process (from t_0_ + 2/8T to t_0_ + 4/8T in Figure 6), but by the end of the feeding process, it became underfilled again. To analyze the cause of the decrease in the number of particles inside Cavity2 during the feeding process, an analysis was performed on the variation in particle numbers in Cavity2 over time. The results of this analysis are shown in Figure 11, relative position of the cavity and feed opening at different stages and their filling levels as shown in Figure 12.

- Stage 1—Empty Phase (pre-feeding): In this stage, the cavity has not yet been fed, and the number of particles inside the cavity remains zero;
- Stage 2—Filling Phase: The cavity moves to the position where it is connected to the feed opening (as shown at t_0_ in Figure 6). Since the cavity has ample space at this stage, particles flow into the cavity rapidly along the cavity gap (FOZ2-Cavity2). This causes a sharp increase in the particle count, as seen in the Figure 11 curve;
- Stage 3—Exchange Balance Phase: In this stage, the cavity is filled. The curve shows a plateau, indicating that the cavity maintains its filled state (As seen in Figure 11). The particle flow observed in Figure 7 shows that particles flow back into the hopper from the upper side of the cavity (FOZ2–VCZ2), while particles from the hopper enter the cavity from the lower side (aft-side) (FOZ2–VEZ2). The inflow and outflow of particles are balanced, maintaining the filled state of the cavity;
- Stage 4—Back Feeding Phase: As the cavity continues to move forward in a spiral, the volume of the VEZ expands against gravity direction. The particles, relying on their weight, cannot flow into the cavity effectively, leading to the formation of a void at the front of the cavity. The decrease in particle count at the early part of this stage is due to the number of particles flowing back from the upper side being greater than the number flowing into the cavity from the lower side, as seen at t_0_ + 4/8T in Figure 7. Later in this stage, after the cavity has fully passed through the feed opening, the decrease in particle count is due to the gap at the advancing face of VCZ2, which causes particles to flow back from the lower side of FOZ2 (FOZ2–VCZ2), as shown with t_0_ + 6/8T in Figure 7;
- Stage 5—Closed Conveying: In this final stage, the cavity becomes disconnected from the feed opening. Since the cavity is now closed, the particles inside the cavity remain in place. The number of particles retained in the cavity during this stage determines the feeding efficiency of the ERE.

### 3.6. Analysis of the Back Feeding Process

**Figure 13:**
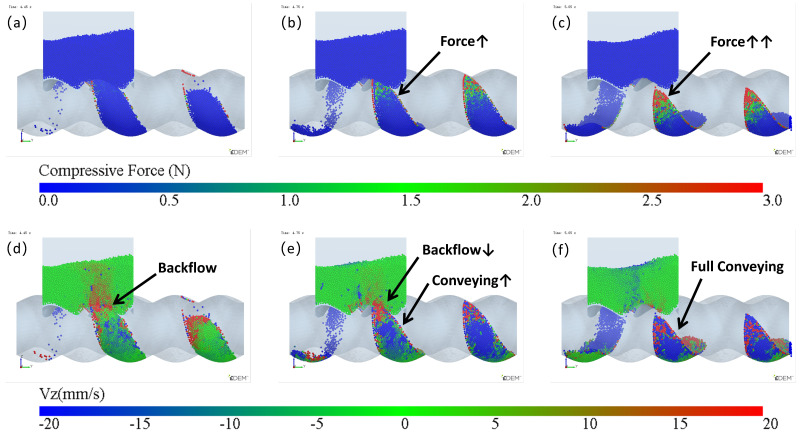
The evolution of particles in the cavity during the late stage of back feeding: (**a**–**c**) compressive force, (**d**–**f**) Vz.

From the analysis of the feeding process, the cavity is already filled during stage3, indicating that the low filling issue of the original structure is not caused by the feed opening being too small or the feed rate being too low. The back feeding in stage4 is a unique phenomenon in the ERE feeding process, and it is key to solving the low-filling problem of the cavity. During the early stages of back feeding, when the cavity passes through the feed opening, particles in the upper side are squeezed back into the hopper due to the advancing face of the cavity. This part of back feeding is difficult to avoid. The particles that remain in the cavity at the end of the process mainly come from the lower side of the cavity. In the later stages of the back feeding, the pressure and velocity (Vz) changes in the lower side cavity are illustrated in Figure 13. From Figure 13a, when the volume in the lower side of the cavity near the feed opening starts to shrink, the particles begin to be compressed, and some particles flow back into the hopper (Figure 13d). As the volume continues to shrink (Figure 13b), the pressure on the particles increases, causing some particles to flow forward along the cavity, while others flow back into the hopper (Figure 13e). Once the cavity is disconnected from the feed opening (Figure 13c), the pressure continues to rise, and since the cavity is no longer connected to the feed opening, the advancing face can push all the particles forward (Figure 13f). Thus, the back feeding in the lower side cavity is primarily caused by the unsealed advancing face. The gap in the advancing face, on one hand, reduces the pressure, making it insufficient to push all the particles forward, and on the other hand, the gap provides a path for the particles to flow back into the hopper under pressure. To reduce the back feeding in the lower side cavity, it is crucial to seal the advancing face as early as possible. Since the cavity moves at a constant speed along the axis when the rotational speed and pitch are fixed, the time it takes for the advancing face to seal is directly proportional to the length of the feed opening. By shortening the feed opening length (the axial opening length of FOZ2), the advancing face can seal earlier, thus increasing the effect of feeding.

### 3.7. Variation of the Feeding Process with Changes in Feeding Opening Length

**Figure 14:**
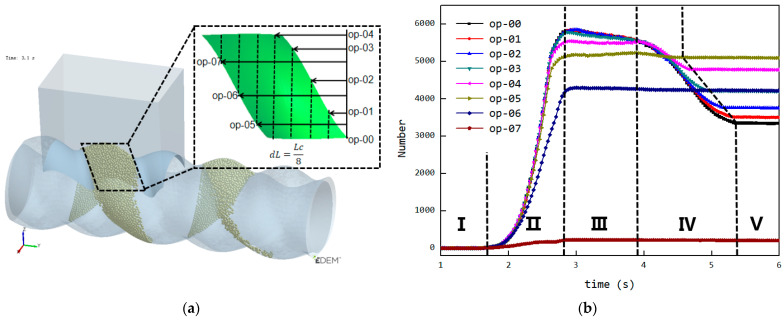
Feed opening structures with different opening length: (**a**) schematic diagram of the structure, (**b**) changes in the number of particles within the cavities during the feeding process.

To verify the effect of shortening the feed opening on reducing back feeding in the cavity, a design was made with eight groups of feed openings having different shortening amounts, as shown in Figure 14a. These feed openings were derived from the original FOZ2 in Figure 9, where the total axial length was Lc (in this case, Lc ≈ 52 mm), with shortening amounts varying from 0/8Lc to 7/8Lc. When simulations were conducted with feed openings of different lengths, the curve showing the variation of the number of particles in the cavity over time is shown in Figure 14b. From the curve, as the feed opening length decreases, and the final stage of the curve (the plateau) shifts earlier, indicating that the fifth stage (closed conveying stage) moves progressively forward. As a result, when the shortening amount changes from 0/8Lc to 5/8Lc, the number of particles retained in the cavity gradually increases. However, when the shortening amount increases further, although the back feeding decreases, the reduction in the feed opening impacts the feeding rate during the second stage (filling phase), leading to a decrease in the number of particles retained in the cavity.

**Figure 15:**
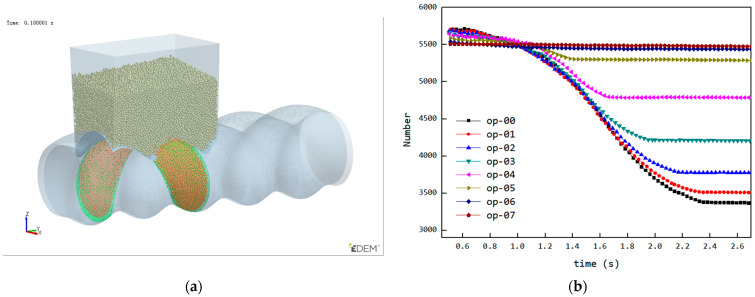
Under the initial setup state: (**a**) initial position of the cavity (the area indicated by the green mesh), (**b**) simulation results.

The reduction in the feed opening length affects both the filling phase and the back feeding phase, which may lead to the reasons for the variation in particle quantity within the cavity being difficult to determine. To analyze how the reduction in feed opening length affects the back feeding, the following initial conditions were set for the simulation (cavity is filled during the third stage): the initial position of the cavity was set as shown in Figure 15a, at the position just before entering the back feeding phase, and the cavity was filled with particles to ensure that at the start of the back feeding phase, the filling condition was the same under different feed opening lengths. The simulation results, showing the particle count in the cavity over time, are presented in Figure 15b. From the curve, it can be observed that the back feeding curves under different feed opening conditions follow a similar downward trajectory, indicating that the back feeding process is controlled via the VCZ compression process, which is primarily governed by the structure and rotational speed of the ERE. As the feed opening length decreases, the end time of the descending curve gradually advances. Due to the earlier end of the back feeding phase, the number of back feeding particles is reduced, leading to an increase in the particle quantity inside the cavity during closed conveying.

**Figure 16:**
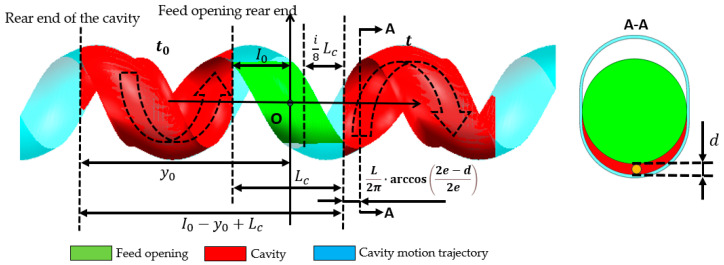
Schematic diagram of theoretical back feeding cutoff time calculation.

**Figure 17:**
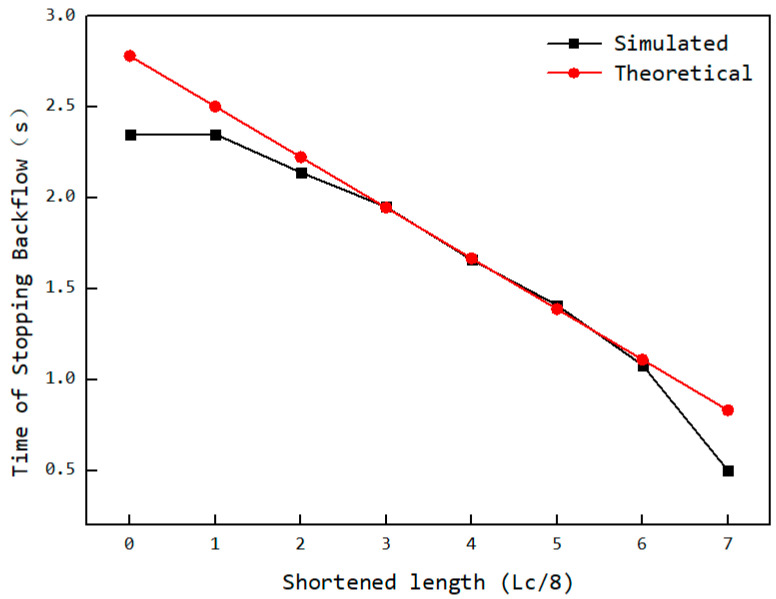
Variation in back feeding cutoff time with the shortened length.

Theoretically, the cutoff time for back feeding is related to the cavity’s connection status with the feed opening (as Figure 16). The initial axial position of the rear end of the cavity is *y*_0_, and the axial position of the rear end of the feed opening is *I*_0_. The axial distance that the cavity needs to move from its initial position at time *t*_0_ until it ceases connection with the feed opening is *I*_0_ − *y*_0_ + Lc. Due to the particle diameter *d*, when the distance between the rotor and stator reaches the position shown in section A–A of Figure 16, the particles in the cavity can no longer flow back into the hopper, thereby shortening the required distance by $\frac{L}{2\pi }\cdot \arccos\left(\frac{2e-d}{2e}\right)$. Additionally, because the feed opening is shortened by an amount of $i\cdot \frac{L_{c}}{8}$, the corresponding distance is further reduced. Given the axial movement speed of the cavity *wL*, the time *t* at which back feeding ends (i.e., when the cavity particles can no longer flow back into the hopper) can be calculated according to Equation (5). The comparison between the theoretical back feeding cutoff time and the simulated results is shown in Figure 17. The curve indicates that, except for the values at both ends being smaller than the theoretical values, the remaining data points align closely with the theoretical calculation. This suggests that the reduction in the feed opening length is approximately proportional to the advancement of the back feeding cutoff time. The deviation at the ends is due to the sharp angular regions at the front and rear of the feed opening area, which, as particle flow channels, are too small, making particle flow difficult. This leads to the closure of the advancing face earlier than expected, causing an earlier cutoff of back feeding.

Theoretical back feeding cutoff time *t*:

(5) $$t=\frac{1}{wL}\cdot \left(I_{0}+L_{c}-i\cdot \frac{L_{c}}{8}-y_{0}-\frac{L}{2\pi }\cdot \arccos\left(\frac{2e-d}{2e}\right)\right)+t_{0},$$

where the following applies: *w* is the revolution velocity [rps]; *L* is the stator pitch [mm]; *I*_0_ is the axial coordinate of the feed opening rear end [mm]; *i* is the feed opening shortening amount; Lc is the initial length of the feed opening [mm]; *y*_0_ is the initial axial coordinate of the rear end of the cavity [mm]; *e* is the eccentric distance [mm]; *d* is the particle diameter [mm]; and *t*_0_—start time of rotor rotation [s].

**Figure 18:**
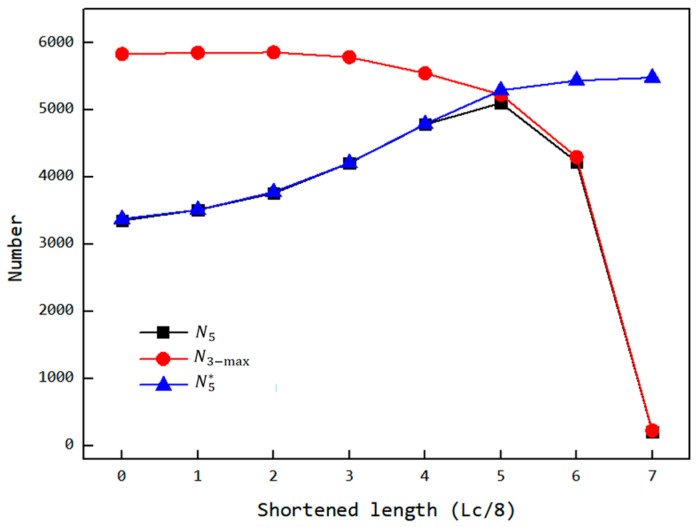
Variation in the number of particles inside the chambers (maximum value in the third stage $N_{3-\max}$, the fifth stage, $N_{5}$ and the fifth stage under the condition of full filling before backflow $N_{5}^{*}$) with the shortened length.

**Figure 19:**
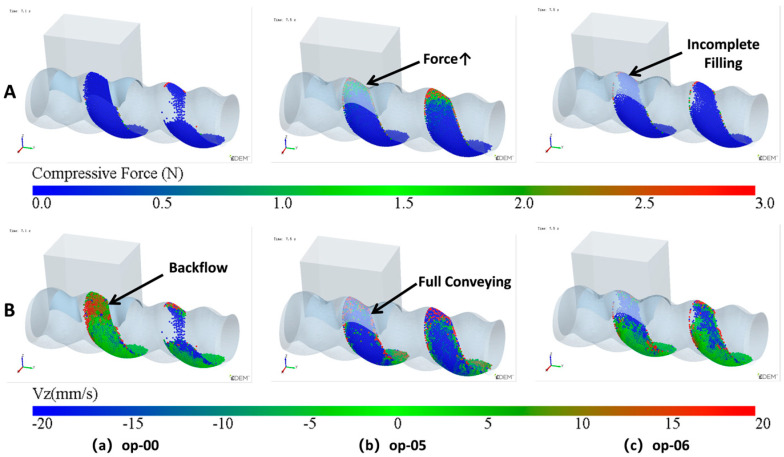
Particle parameters during the backflow stage: A—compressive force, B—Vz: (**a**) unoptimized feed opening, op-00; (**b**) appropriately feed opening length, op-05; (**c**) overly small feed opening, op-06.

As shown in Figure 18, under normal simulation conditions, the number of particles inside the cavity (maximum value in the third stage $N_{3-\max}$ and the fifth stage $N_{5}$), as well as the number of particles inside the cavity when it is initially filled before the back feeding stage (fifth stage $N_{5}^{*}$), varies with the reduction in the feed opening length. From the trend of the curves, it can be observed that if the effect of the reduced feed opening on the filling stage is ignored, and the cavity is kept filled before the back feeding stage, $N_{5}^{*}$ increases monotonically with the reduction in the opening length. This is because the back feeding stop time is advanced. Under normal simulation conditions, the trend of the number of $N_{5}$ follows an initial increase and then a decrease with the reduction in the feed opening length. Between op-00 and op-05, $N_{5}$ increases as the feed opening length decreases, with the growth curve overlapping the curve of $N_{5}^{*}$. Between op-05 and op-07, however, $N_{5}$ decreases with the reduction in the feed opening length, and the curve deviates from the curve of $N_{5}^{*}$ while still overlapping with the curve of $N_{3-\max}$. This behavior occurs because when the filling amount decreases, the number of particles in the cavity during the back feeding phase is insufficient to fully fill the VCZ (as shown in Figure 19c). The compression of the VCZ does not generate enough pressure to move the particles, and the number of particles in the cavity remains unchanged, similar to $N_{3-\max}$.

Therefore, $N_{5}^{*}$ somewhat reflects the ability of the VCZ to accommodate particles when the back feeding ends. The earlier the back feeding stop time, the larger the VCZ volume, and the greater the ability to accommodate particles. When $N_{3-\max}>N_{5}^{*}$, the number of particles exceed the capacity of the VCZ, compression of the VCZ forces the excess particles to flow out, leading to $N_{5}=N_{5}^{*}$. When $N_{3-\max}<N_{5}^{*}$, the VCZ can accommodate all particles, back feeding does not occur, leading to $N_{5}=N_{3-\max}$. From the curve’s trend, $N_{3-\max}$ decreases with the increase in the feed opening reduction, and $N_{5}^{*}$ increases with the reduction in the opening length. The maximum value of $N_{5}$ will appear at the intersection of the two curves, which occurs at a reduction in 5/8Lc, as shown in Figure 18.

### 3.8. Optimization Principle and Design of the Optimized Structure

The principle of how the feeding effect changes with the feed opening length is shown in Figure 19. When the feed opening is not optimized, as shown in Figure 19a, as previously mentioned, the absence of a sealed advancing surface causes some particles to flow back into the hopper. When the feed opening is appropriately sized, as shown in Figure 19b, the feed opening is sealed early, forming an effective advancing surface in the VCZ. The particles filled during the filling stage are just enough to fill the VCZ at this point. The closure of the cavity and the advancing surface provide sufficient pressure, and most of the particles flow forward. When the feed opening is further reduced, as shown in Figure 19c, the sealing of the advancing surface occurs even earlier, reducing the back feeding. However, when the feed opening is too small, the amount of filling during the filling stage becomes too low, leading to a decrease in the final number of particles retained in the cavity.

**Figure 20:**
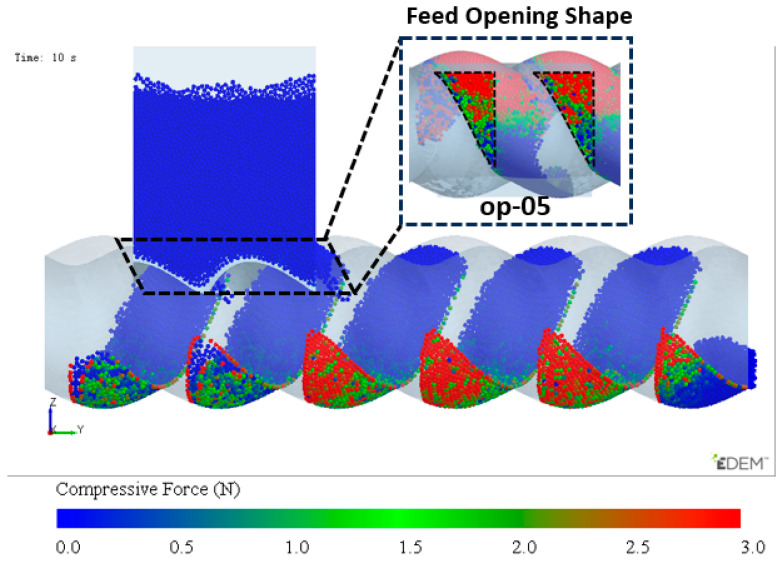
Optimized opening design scheme for dual-cavity adaptation and feeding effect.

From the above analysis, it can be concluded that the feed opening structure op-05 has the optimal feeding capacity. Combined with the DCBD design scheme, a new feed opening structure was designed as shown in Figure 20. And the feeding effect of this structure shows that not only is the filling of the dual-cavity uniform, but also, compared to Figure 9, the filling degree is significantly improved. The optimized structure shows a remarkable improvement in performance.

**Figure 21:**
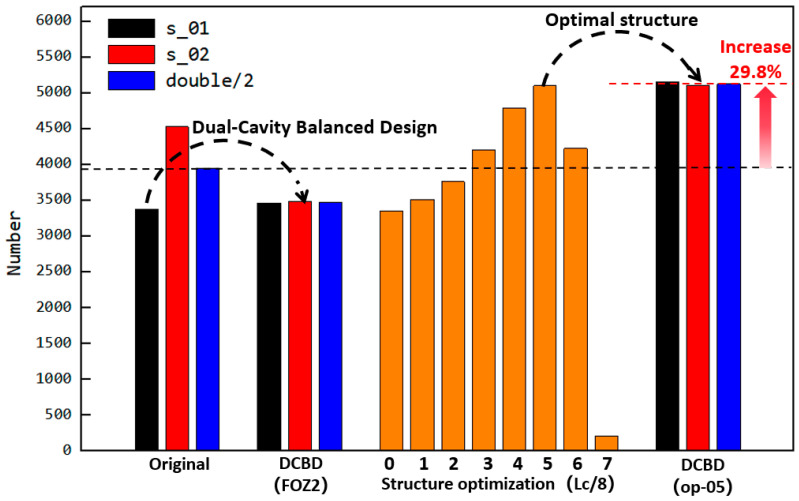
Influence of feed openings with different structures on the filling quantity (the orange bars indicate the effect of the optimized feed opening.).

The impact of different feed opening structures on filling amounts is summarized in Figure 21. Under the original structure, the filling degree of the dual-cavity (s_01 and s_02) is uneven. Through DCBD with the feed opening structure FOZ2, the filling amount of both cavities is balanced. Reducing the opening length of FOZ2 and further optimizing led to the conclusion that structure op-05 provides the optimal feeding effect. With the dual-cavity adaptation of the optimal feed opening structure op-05, not only is the dual-cavity filling balanced, but the filling amount is also increased by 29.8%.

## 4. Experimental Validation

**Figure 22:**
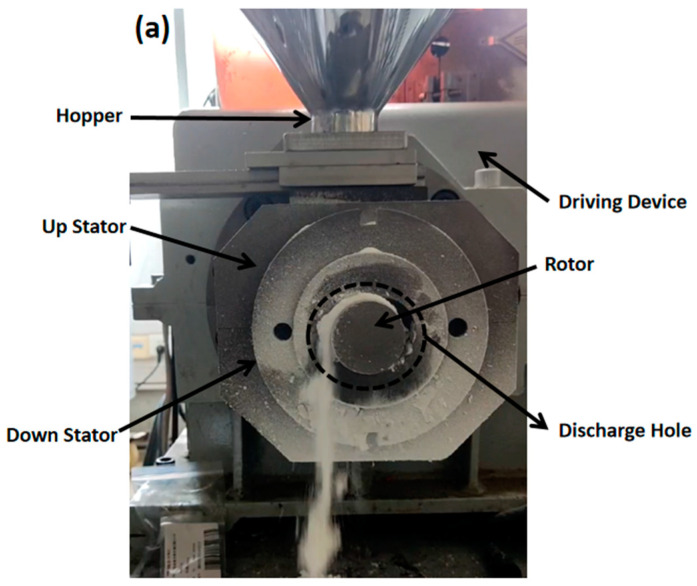

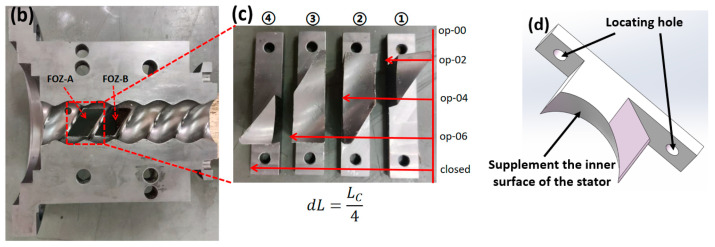
Adjustable feed opening experimental machine: (**a**) components of the adjustable feed opening device, (**b**) upper stator and feed opening, (**c**) feed opening profile accessory, (**d**) adjustment accessories part ②.

To verify the feasibility and mechanism of the optimization plan, verification equipment (the main structural parameters of the stator and rotor are shown in Table 1) was designed as shown in Figure 22. The equipment includes feed ports (FOZ-A and FOZ-B) located at the corresponding positions (similar to the feed port position in Figure 8), along with adjustment accessories (used to supplement the inner surface of the stator and shorten the feed opening) (①–④) for altering the feed port opening. The width of the accessories is 1/4Lc. When no accessories are installed, it corresponds to the simulation of op-00. As the accessories (①–④) are added sequentially, they correspond to op-02, op-04, op-06, and the closed state. The exploded view of the equipment is provided in Supplementary Material Video S3. The material used in the experiment is ultra-high-molecular weight polyethylene UPE4040.

**Figure 23:**
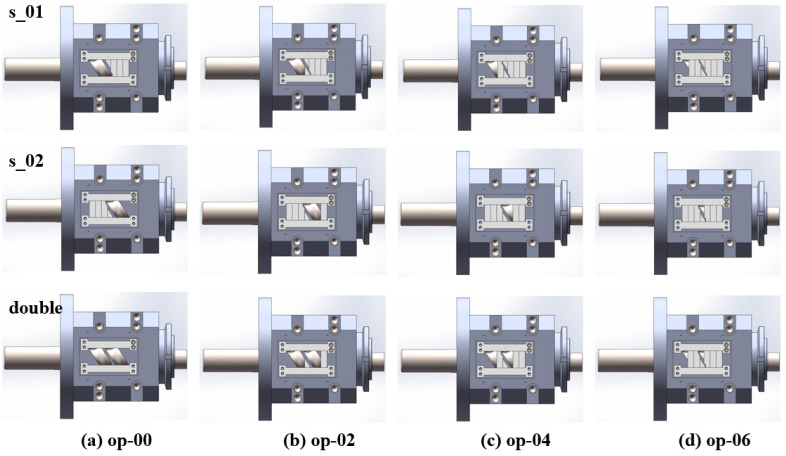
Different component configurations in the experiment: (**a**) op-00, (**b**) op-02, (**c**) op-04, (**d**) op-06.

**Figure 24:**
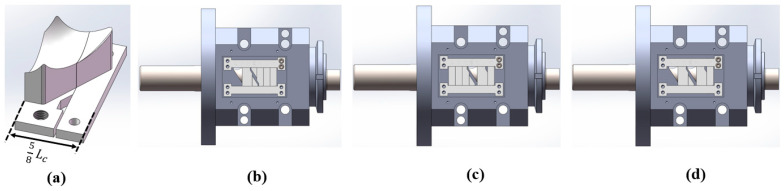
Accessories and the ERE feed port configuration of op-05: (**a**) accessories, (**b**) s_01, (**c**) s_02, (**d**) double.

The following experiments were conducted, and the corresponding structural component configuration is shown in Figure 23:

- Single feed into FOZ-A: measured the output (s-01) under different configurations (without parts, and with parts ①, ②, ③ corresponding to op-00, op-02, op-04, op-06);
- Single feed into FOZ-B: measured the output (s-02) under the same configurations;
- Simultaneous feed into FOZ-A and FOZ-B: measured the output (double/2) when both ports were fed simultaneously under different configurations.
- In addition, to validate the performance of op-05, the designed accessories and the ERE feed port configuration are illustrated in Figure 24.

Finally, the experimental results were compared with simulation results by converting the output into volumetric throughput.

**Figure 25:**
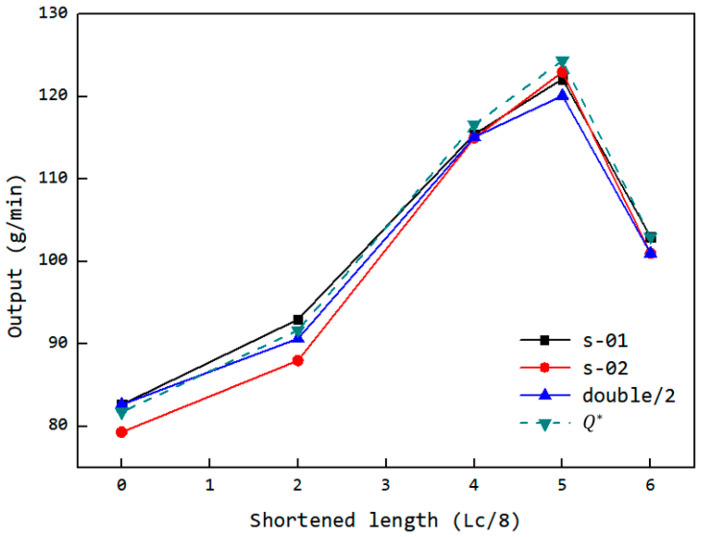
Variation of the output with the feed opening shortening amount and the simulated equivalent output of the ERE adjustable feed opening experimental machine (s-01: single feeding for FOZ-A; s-02: single feeding for FOZ-B; double: simultaneous feeding).

The corresponding output results are shown in Figure 25. The simulation results indicate that the uneven filling of the dual cavity was due to the lack of adapted feed port structures, which led to the proposed solution of dual-cavity adaptation. The experiment achieved dual-cavity adaptation by installing equal profile components in both FOZ-A and FOZ-B. As seen from the changes in the s-01 and s-02 curves, after adaptation, the outputs of s-01 and s-02 became similar, demonstrating that setting equal feed port structures is effective in balancing the feed into the cavities. On the other hand, the installation of profile components at different positions allowed control over the feed port opening. The output variation with shortening amounts, as shown in Figure 25, follows a curve that first increases and then decreases, consistent with the simulation results. Adjusting the feed port opening is an effective method for enhancing the feed capacity of the ERE.

To better compare the simulation and experimental results, the equivalent UPE4040 output based on volume yield from the simulation was calculated (according to Equations (6) and (7)), as shown in Figure 25. It can be observed that there is a good correspondence between the simulation and experimental results. While the material properties in the simulation differ from those of the UPE4040 used in the experiment, the volume-based simulation results still effectively predict the experimental phenomena. This suggests that the feedback process in the ERE is driven primarily by the volume change during VCZ compression and is less sensitive to variations in material properties. Therefore, the structural design of the ERE plays a crucial role in regulating the feed performance.

The equivalent UPE4040 output Q*:

(6) $$Q_{v}=N\cdot \frac{V}{N_{max}},$$

(7) $$Q^{*}=w\cdot \rho \cdot Q_{v}$$

where the following applies: *Q_v_* is the filling degree of particles in the cavity [mm^3^]; *N* is the number of particles inside the cavity; *V* is the volume of the cavity [mm^3^]; *N_max_* is the number of particles inside the filled cavity; Q* is the equivalent throughput of UHMWPE 4040 [g/s]; *w* is the rotational speed [r/s]; *ρ* is the loose bulk density of UHMWPE 4040 [g/mm^3^].

## 5. Conclusions

This work used discrete element method (DEM) simulation technology to model the solid conveying process in the ERE. Based on visual analysis of the positive displacement conveying process and analysis of the output parameters, a design solution for addressing the issues of extrusion instability and low fill level in the ERE was proposed. The optimization effects of the design were validated through both simulations and experiments.

First, through DEM simulations of the solid conveying section of the ERE, the conveying process was visualized, which allowed the identification of the key process affecting the cavity fill level—the feeding process. Second, based on the visual analysis of the feeding process and considering the specific regions of dual-cavity feeding, a feeding opening design solution was proposed to address the issue of imbalanced filling in the dual-cavity: dual-cavity balanced design (DCBD). Third, by analyzing the back feeding process in a relatively empty cavity, it was found that the duration of back feeding is related to the length of the corresponding feed opening. A structural optimization solution was proposed to reduce the length of the feed opening. The simulation results showed that reducing the feed opening length significantly improved the feeding effect without affecting the number of particles filled during the filling phase. When the length reduction reached 5/8Lc, the fill level was just enough to fill the VCZ, achieving the maximum reduction in length while ensuring optimal feeding performance. The simulation results show that, compared with the original structure, the optimized dual-feed opening structure increases the feed capacity from 3953 particles per cavity to 5132 particles per cavity, an improvement of 29.8%, and achieves balanced filling between the two cavities. Finally, experimental validation indicates that the UPE4040 output can be increased from 165.3 g/min with structure op-00 to 231.7 g/min with the optimized structure op-05.

The main work of this paper focuses on the results obtained under specific structural configurations of the ERE, as well as specific rotational speeds and material property conditions. In the future, a study will investigate the feeding behavior under different conditions: material properties, structural parameters (such as pitch, diameter, and eccentricity), and rotational speed. The effectiveness of the optimized feed opening under various conditions will be analyzed, aiming to further extend the proposed optimization method for the feed opening. This will provide broader guidance for the inlet design of ERE. Through these works, the feeding amount will be predicted, providing guidance for the design of the compression ratio in the plasticizing and transport section of the ERE. The optimization of the feed opening structure improves the output, helps shorten the length-to-diameter ratio of the ERE, increases capacity, and reduces energy consumption.

## Figures and Tables

**Table 1:** Geometric structure and operating parameters.

Parameters	Value
Diameter of stator semi-circular segments *D*_1_ (mm)	40
Rotor diameter *D*_2_ (mm)	40
Rotor eccentricity *e* (mm)	3
Length of stator straight segments 4*e* (mm)	12
Pitch *L*_1_ (mm)	70
Rotor pitch *L*_2_ (mm)	35
Revolution velocity *w* (rpm)	20
Rotational velocity *w*_1_ (rpm)	−40

**Table 2:** Input parameters used to develop the simulations.

Parameters	Value
Particle diameter (mm)	0.7
Particle Poisson’s ratio	0.4
Particle shear modulus (Pa)	10^8^
Particle density (kg/m^3^)	1500
Geometry Poisson’s ratio	0.29
Geometry shear modulus (Pa)	7.992 × 10^10^
Geometry density (kg/m^3^)	7861
Particle-particle restitution coefficient	0.45
Particle-particle coefficient of static friction	0.5
Particle-particle coefficient of rolling friction	0.05
Particle-geometry restitution coefficient	0.2
Particle-geometry coefficient of static friction	0.5
Particle-geometry coefficient of rolling friction	0.01
Time step (s)	2.26077 × 10^−6^
Simulation time (s)	6–12

**Figure A1:**
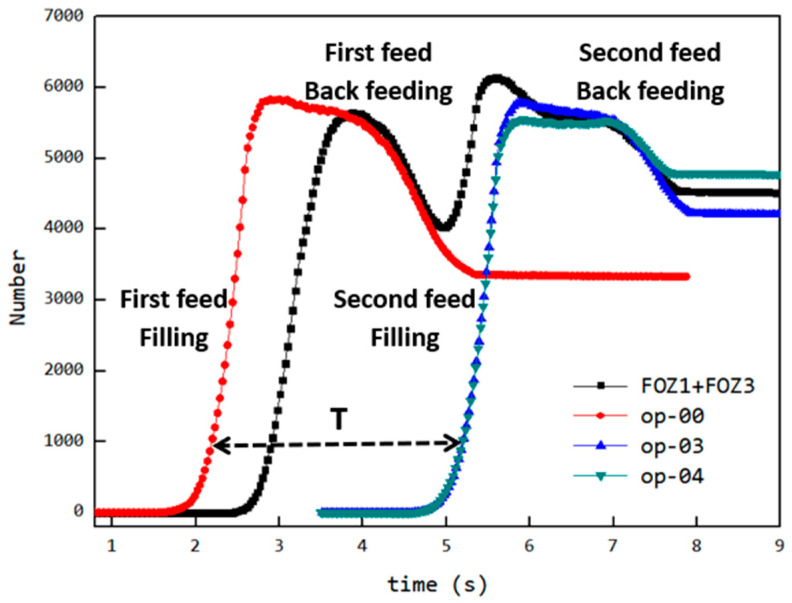
Analysis of two feeding processes in fuller cavity.

## Data Availability

The original contributions presented in this study are included in the article/Supplementary Material. Further inquiries can be directed to the corresponding authors.

## Supplementary Materials

The following supporting information can be downloaded at https://www.mdpi.com/article/10.3390/ma18091939/s1, Video S1: ERE solid conveying process; Video S2: Variation in the vertical velocity near the feed opening; Video S3: Exploded view; Python code S1: Cavity particle numbers count.

## Abbreviations

The following abbreviations are used in this manuscript:

ERE	Eccentric rotor extruder
DEM	Discrete element method
VCZ	Volume compression zone
VEZ	Volume extension zone
FOZ	Feed opening zone
DCBD	Dual-cavity balanced design

## Appendix A

The reason why Cavity1 and Cavity3 are fuller compared to Cavity2 in the original structure is the shorter length of the feed opening area (FOZ3) compared to FOZ2. As shown in Figure A1, the feeding curves for the first and second feed processes are compared with the curves for op-00, op-03, and op-04. From the changes in the curves in Figure A1, during the first feeding, although the feed opening was reduced, the decrease in opening from the rear to the front not only failed to reduce back feeding but also delayed the start of the filling stage, thereby lowering the filling speed. The curve during the back feeding stage is consistent with op-00, without improvement. During the second feeding, the feed opening (FOZ3) was shortened by 3.65/8Lc, which was between op-03 and op-04. From the curve changes, although the cavity was already more filled during the second feeding, allowing it to fill faster, the curve during the back feeding stage was similar to op-03 and op-04. In the end, the number of particles remaining at the end of the closure phase was between op-03 and op-04. This indicates that the better filling performance of the cavity is primarily determined by the feed opening (FOZ3) during the second feeding and was achieved by reducing the length of the feed opening to decrease back feeding, thus enabling the cavity to achieve a higher filling degree.

## References

[1] Yuan Z., Chen X., Yu D. (2021). Recent advances in elongational flow dominated polymer processing technologies. Polymers.

[2] He G., Lin Z., Yin X., Feng Y. (2022). High orientation structure in UHMWPE/BN composites continuously obtained by elongational flow field leading to superior thermal conductivity. J. Appl. Polym. Sci..

[3] Wu T., Tong Y., Qiu F., Yuan D., Zhang G., Qu J. (2018). Morphology, rheology property, and crystallization behavior of PLLA/OMMT nanocomposites prepared by an innovative eccentric rotor extruder. Polym. Adv. Technol..

[4] Yu X., He J., Liu Y., Su L., Liu F. (2019). Structure-property relationship of polycarbonate/polypropylene alloys prepared: Via eccentric rotor extruder. RSC Adv..

[5] He H.Z., Xue F., Jia P.F., He G.J., Huang Z.X., Liu S.M., Xue B. (2018). Linear low-density polyethylene/poly(ethylene terephthalate) blends compatibilization prepared by an eccentric rotor extruder: A morphology, mechanical, thermal, and rheological study. J. Appl. Polym. Sci..

[6] Zhang H., Wei X., Qu J.P. (2021). Microstructure evolution and mechanism of PLA/PVDF hybrid dielectrics fabricated under elongational flow. Polymer.

[7] Feng Y., Gao Y., Chen J., Jiang J., Yin X., He G., Zeng Y., Kuang Q., Qu J. (2019). Properties of compression molded ultra-high molecular weight polyethylene products pretreated by eccentric rotor extrusion. Polym. Int..

[8] Lin W., Hou A., Feng Y.H., Yang Z.T., Qu J.P. (2019). UHMWPE/organoclay nanocomposites fabricated by melt intercalation under continuous elongational flow: Dispersion, thermal behaviors and mechanical properties. Polym. Eng. Sci..

[9] Qu J., Zhang G., Yin X. (2019). Volume Pulsed Deformation Plasticating and Conveying Method and Device by Eccentric Rotor. US Patent.

[10] Cundall P.A., Strack O.D.L. (1979). A discrete numerical model for granular assemblies. Géotechnique.

[11] Hu G., Chen J., Jian B., Wan H., Liu L. Modeling and simulation of transportation system of screw conveyors by the discrete element method. Proceedings of the 2010 International Conference on Mechanic Automation and Control Engineering, MACE2010.

[12] Pezo L., Jovanović A., Pezo M., Čolović R., Lončar B. (2015). Modified screw conveyor-mixers—Discrete element modeling approach. Adv. Powder Technol..

[13] Orefice L., Khinast J.G. (2017). DEM study of granular transport in partially filled horizontal screw conveyors. Powder Technol..

[14] Wang S., Li H., Tian R., Wang R., Wang X., Sun Q., Fan J. (2019). Numerical simulation of particle flow behavior in a screw conveyor using the discrete element method. Particuology.

[15] Yu W., Zou D., Li D., Wang Q., Peng P. (2024). Development of Models Relating Screw Conveying Capacity of Concrete to Operating Parameters and Their Use in Conveyor Operating Strategies to Consider Batch Production. Appl. Sci..

[16] Moysey P.A., Thompson M.R. (2005). Modelling the solids inflow and solids conveying of single-screw extruders using the discrete element method. Powder Technol..

[17] Moysey P.A., Thompson M.R. (2004). Investigation of solids transport in a single-screw extruder using a 3-D discrete particle simulation. Polym. Eng. Sci..

[18] Fernandez J.W., Cleary P.W., McBride W. (2011). Effect of screw design on hopper drawdown of spherical particles in a horizontal screw feeder. Chem. Eng. Sci..

[19] Kretz D., Callau-Monje S., Hitschler M., Hien A., Raedle M., Hesser J. (2016). Discrete element method (DEM) simulation and validation of a screw feeder system. Powder Technol..

[20] Han Y., Jia F., Zeng Y., Jiang L., Zhang Y., Cao B. (2017). DEM study of particle conveying in a feed screw section of vertical rice mill. Powder Technol..

[21] Norouzi H.R., Zarghami R., Mostoufi N. (2015). Insights into the granular flow in rotating drums. Chem. Eng. Res. Des..

[22] Wu W.N., Liu X.Y., Zhang R., Hu Z. (2019). DEM investigation of the power draw for material movement in rotary drums with axis offset. Chem. Eng. Res. Des..

[23] Hu Z., Liu X., Wu W. (2018). Study of the critical angles of granular material in rotary drums aimed for fast DEM model calibration. Powder Technol..

[24] Liu X., Hu Z., Wu W., Zhan J., Herz F., Specht E. (2017). DEM study on the surface mixing and whole mixing of granular materials in rotary drums. Powder Technol..

[25] Wang X., Yi J., Zhou Z., Yang C. (2020). Optimal speed control for a semi-autogenous mill based on discrete element method. Processes.

[26] Golshan S., Zarghami R., Norouzi H.R., Mostoufi N. (2017). Granular mixing in nauta blenders. Powder Technol..

[27] Göbel F., Golshan S., Norouzi H.R., Zarghami R., Mostoufi N. (2019). Simulation of granular mixing in a static mixer by the discrete element method. Powder Technol..

[28] Mateo-Ortiz D., Méndez R. (2016). Microdynamic analysis of particle flow in a confined space using DEM: The feed frame case. Adv. Powder Technol..

[29] Ramírez-Aragón C., Ordieres-Meré J., Alba-Elías F., González-Marcos A. (2020). Numerical modeling for simulation of compaction of refractory materials for secondary steelmaking. Materials.

[30] Bembenek M., Buczak M., Baiul K. (2022). Modelling of the Fine-Grained Materials Briquetting Process in a Roller Press with the Discrete Element Method. Materials.

[31] Owen P.J., Cleary P.W. (2009). Prediction of screw conveyor performance using the Discrete Element Method (DEM). Powder Technol..

[32] Li X., Hou Q., Dong K., Zou R., Yu A. (2020). Promote cohesive solid flow in a screw feeder with new screw designs. Powder Technol..

[33] Dun G., Sheng Q., Ji X., Zhang C., Gao S., Wei Y., Han Y. (2024). Optimal Design and Experiment of Electronically Controlled Inclined Spiral Precision Fertilizer Discharger. Agriculture.

[34] Fu L., Wang K., Hu B., van Xo N. (2021). Conveying performance of drilling tool of auger miner at different strike angles based on discrete element method. Powder Technol..

[35] Mindlin R.D. (1949). Compliance of Elastic Bodies in Contact. J. Appl. Mech..

[36] Jayasundara C.T., Yang R.Y., Yu A.B., Curry D. (2008). Discrete particle simulation of particle flow in IsaMill Effect of grinding medium properties. Chem. Eng. J..

